# Monitoring human enteric viruses in wastewater and relevance to infections encountered in the clinical setting: a one-year experiment in central France, 2014 to 2015

**DOI:** 10.2807/1560-7917.ES.2018.23.7.17-00237

**Published:** 2018-02-15

**Authors:** Maxime Bisseux, Jonathan Colombet, Audrey Mirand, Anne-Marie Roque-Afonso, Florence Abravanel, Jacques Izopet, Christine Archimbaud, Hélène Peigue-Lafeuille, Didier Debroas, Jean-Luc Bailly, Cécile Henquell

**Affiliations:** 1 Université Clermont Auvergne, CNRS, Laboratoire Microorganismes: Génome et Environnement, Clermont-Ferrand, France; 2 CHU Clermont-Ferrand, Centre National de Référence Entérovirus et parechovirus - Laboratoire Associé, Laboratoire de Virologie, Clermont-Ferrand, France; 3 AP-HP, Hôpital Paul Brousse, Centre National de Référence Virus des hépatites à transmission entérique (hépatite A) - Laboratoire Associé, Laboratoire de Virologie, Villejuif, France; 4 CHU Toulouse, Centre National de Référence Virus des hépatites à transmission entérique (hépatite E) - Laboratoire Coordonnateur, Laboratoire de Virologie, Toulouse, France

**Keywords:** sewage, enteric viruses, Epidemiology, clinical and environmental surveillance, wastewater, EV-D68, hepatitis A virus, hepatitis E virus

## Abstract

Human enteric viruses are resistant in the environment and transmitted via the faecal-oral route. Viral shedding in wastewater gives the opportunity to track emerging pathogens and study the epidemiology of enteric infectious diseases in the community. **Aim:** The aim of this study was to monitor the circulation of enteric viruses in the population of the Clermont-Ferrand area (France) by analysis of urban wastewaters. **Methods:** Raw and treated wastewaters were collected between October 2014 and October 2015 and concentrated by a two-step protocol using tangential flow ultrafiltration and polyethylene glycol precipitation. Processed samples were analysed for molecular detection of adenovirus, norovirus, rotavirus, parechovirus, enterovirus (EV), hepatitis A (HAV) and E (HEV) viruses. **Results:** All wastewater samples (n = 54) contained viruses. On average, six and four virus species were detected in, respectively, raw and treated wastewater samples. EV-positive samples were tested for EV-D68 to assess its circulation in the community. EV-D68 was detected in seven of 27 raw samples. We collected data from clinical cases of EV-D68 (n = 17), HAV (n = 4) and HEV infection (n = 16) and compared wastewater-derived sequences with clinical sequences. We showed the silent circulation of EV-D68 in September 2015, the wide circulation of HAV despite few notifications of acute disease and the presence in wastewater of the major HEV subtypes involved in clinical local cases. **Conclusion:** The environmental surveillance overcomes the sampling bias intrinsic to the study of infections associated with hospitalisation and allows the detection in real time of viral sequences genetically close to those reported in clinical specimens.

## Introduction

Environmental surveillance of specimens contaminated by human faeces is used to monitor enteric virus transmission in the population. It is crucial for monitoring water quality, because enteric viruses excreted in the environment are a potential source of human contamination and community-wide outbreaks [1-3]. The World Health Organization (WHO) added environmental surveillance of poliovirus to that of acute flaccid paralysis in the strategy plan of the Global Polio Eradication Initiative [4]. A recent study in Israel showed that this method was effective in detecting and investigating the silent introduction of wild polioviruses in a polio-free country [5]. Environmental surveillance of urban wastewater can also be useful in tracking emerging viral pathogens and monitoring the changing epidemiology of enteric infectious diseases.

Human enteric viruses include various genera such as adenovirus (ADV), enterovirus (EV), parechovirus (PeV), norovirus (NoV), rotavirus (RV), hepatitis A (HAV) and E (HEV) viruses. They replicate in the gastrointestinal tract and are excreted in large quantities in faeces for several weeks, regardless of whether the infections are symptomatic or not. They are highly resistant in the environment and transmitted via the faecal-oral route following exposure to drinking water, recreational waters and foods contaminated by wastewater and effluents from wastewater treatment plants (WWTP). Enteric viruses are mainly involved in subclinical infections but are also associated with a wide range of symptomatic diseases, including acute gastroenteritis, acute hepatitis, central nervous system infections (meningitis, encephalitis, and paralysis), conjunctivitis and respiratory diseases. In Europe, seroprevalence of hepatitis A and the number of acute HAV infections have declined [6]. In contrast, HEV, which was initially reported as an imported infection, is now recognised as an endemic disease with an increasing number of laboratory-confirmed cases during the last decade [7]. Since mid-2014, enterovirus D68 (EV-D68) has been reported as an emerging pathogen associated with severe respiratory and neurological diseases [8-10].

The main objective of this one-year pilot study was to develop an efficient and rapid method for the prospective detection of a large panel of viral pathogens and to compare them with viral isolates from clinical specimens. The secondary aim was to use the method for epidemiological monitoring and a better understanding of virus circulation.

## Methods 

### Wastewater sampling

From October 2014 to October 2015, wastewater was sampled every two weeks at the WWTP of the urban area of Clermont-Ferrand (Central France) that serves a population of 250,000 inhabitants. The wastewater treatment is based on an activated sludge process with aerobic/anaerobic transition, clarification with ferric chloride to precipitate phosphates, and discharge of the effluent into a local river. The full treatment cycle takes ca 24 hours. Samples of raw (20 L) and treated wastewater (50 L) were collected 24 hours apart at the entry point and the effluent level, respectively, and filtered under gravity through a 50 µm nylon fibre filter. The samples were transferred to the laboratory within 1 hour after collection and processed immediately. A total of 54 samples, 27 each from the influent wastewater and the effluent water, were collected at the Clermont-Ferrand WWTP over the study period.

### Virus concentration

Virus concentration was based on a two-step concentration (tangential flow ultrafiltration and polyethylene glycol) of large volumes of wastewater collected in the WWTP. After a second gravity filtration stage (20 µm nylon fibre filters), wastewater samples were concentrated by tangential flow ultrafiltration (KrosFlo MiniKros, Spectrum, the Netherlands) using a hollow fibre filter (molecular cut-off: 30 kDa, inner diameter: 200 µm, total surface area: 2.6 m2) and a transmembrane pressure of 0.07 to 0.1 bars. After the addition of 10 mM sodium pyrophosphate decahydrate, samples were shaken at room temperature for 40 min and then sonicated three times (1 min each) in a water bath. Samples were centrifuged at 8,000 × g for 20 min. The pH of supernatants was neutralised to pH 7. Samples were further concentrated by polyethylene glycol (PEG) precipitation. Briefly, PEG 8000 and sodium chloride were added to the supernatant at a final concentration of, respectively, 10% and 0.6% (w/v) and incubated at 4 °C for 24–48 hours. The white phase containing viruses was centrifuged at 8,000 × g at 4 °C for 20 min. Pellets were suspended in three times their volume (range: 20–150 mL) in buffer (0.1 M NaCl, 8 mM MgSO_4_-7H_2_O, 50 mM Tris-HCl and 0.005% (w/v) glycerol, pH 7). After addition of 1 M KCl, the mixture was incubated on ice for 40 min to slowly precipitate PEG in the solution, leaving purified virus particles in suspension. After centrifugation (12,000 × g, 10 min at 4 °C), the supernatant was stored at −20 °C until use.

The raw and treated wastewater samples were concentrated to an average of 60 and 16.5 mL, respectively (concentration factors 333 and 3,000).

### Virus detection by molecular methods

Total nucleic acids were extracted from virus concentrates (1 mL) with the NucliSENS EasyMAG platform (bioMérieux, Marcy l’Etoile, France) using the specific B protocol with 100 µL of silica and an elution volume of 50 µL. An internal control specific to each PCR was added to samples before extraction to assess the efficacy of the extraction and purification procedures and to detect the presence of potential amplification inhibitors. Viral targets were detected with real-time RT-PCR or real-time PCR commercial assays. The limits of detection or quantification for each PCR assay are noted in Table 1. Quantitative assays were available only for ADV and EV. EV quantification was performed with an in-house RT-qPCR [11]. All processed samples that tested negative for EV RNA were analysed with a commercial qualitative RT-PCR assay (Enterovirus@ceeram, bioMérieux, France). Negative and positive controls were included in all PCR reactions; standard precautions were taken to prevent cross-contamination.

**Table 1:** PCR assays used to detect or quantify all enteric viruses studied, Clermont-Ferrand, 2014–2015

Virus	Real-time assay	Detection	Limit of detection
Adenovirus	RealStar Adenovirus PCR Kit 1.0 (Altona)	Quantitative	1.09 copies/μL
Enterovirus	In-house assay [11]	Quantitative	6 copies/µL
	Enterovirus@ceeramTools (Ceeram)	Qualitative	NA
Parechovirus	Parechovirus r-gene (bioMérieux)	Qualitative	0.62 TCID50/mL (PeV1) 9.96 TCID50/mL (PeV2)
Rotavirus	RealStar Rotavirus PCR Kit 1.0 (Altona)	Qualitative	1.2 copies/μL
Norovirus (GI, GII)^a^	RealStar Norovirus PCR Kit 2.0 (Altona)	Qualitative	GI: 0.33 copies/μL GII: 0.25 copies/μL
Hepatitis A virus	RealStar HAV RT-PCR Kit 1.0 (Altona)	Qualitative	0.46 IU/μL
Hepatitis E virus	RealStar HEV RT-PCR Kit 1.0 (Altona)	Qualitative	0.31 IU/μL

NA: not available from the manufacturer.

^a^ Test allowing differentiation between genogroup I and genogroup II.

### Specific detection of enterovirus D genome

All wastewater samples positive for EV RNA were screened with a nested RT-PCR assay and primers designed specifically to detect EV-D genomes. Reverse transcription was done with SuperScript III reverse transcriptase (Invitrogen, France) and random hexanucleotides. The complete VP1 capsid protein gene was amplified with Taq DNA polymerase (Qiagen, France) and primers manually designed from an alignment of all EV-D genomes available in GenBank. A first-round PCR assay was performed with primers EVD-1D_S1 5’-GHGGVTCDWGYCCRACARMYAG-3’ and EVD-1D_R1 5’-AYTGRATHCCWGGVCCYTCRA-3’ under the following conditions: 2 min at 94 °C, followed by 40 cycles of 15 s at 94 °C, 50 s at 54 °C, 1 min 10 at 72 °C and a final step of 5 min at 72 °C. The second-round PCR assay was performed with primers EVD-1D_S2 5’-GCHAAYGTKGGNTAYGTNACHTG-3’ and EVD-1D_R2 5’-TGRTGYYTHCCATGRGCRGCHA-3’ for 2 min at 94 °C followed by 40 cycles of 15 s at 94 °C, 50 s at 50 °C, 52 s at 72 °C and a final step of 5 min at 72 °C.

The PCR products were examined by standard agarose gel electrophoresis and purified before nucleotide sequencing.

### Clinical samples

We focused on HAV, HEV and EV-D68, more frequently associated with severe symptoms requiring hospitalisation. During the study period (October 2014 to October 2015), we collected data from all clinical cases of EV-D68, HAV and HEV infection diagnosed in the University Hospital of Clermont-Ferrand. Seventeen patients (median age: 3.6 years; range: 1 month–71 years) were admitted for respiratory manifestations and were diagnosed with EV-D68 infections. In addition, we examined cases of acute symptomatic hepatitis A (four patients, median age: 26.5 years) and hepatitis E (16 patients, median age: 53 years) detected during the study period at the same hospital. These patients had elevated aminotransferase levels (ALT > 61 UI/L) and specific IgM antibodies (HAVAb M ARCHITECT, Abbott, HEV-IgM WANTAI, Eurobio).

### Sequencing of enterovirus D, hepatitis A and E viruses 

As members of the French National Reference Centres (NRC) for national surveillance of HEV, HAV and EV infections, we prospectively genotyped all clinical strains. Wastewater samples were sequenced in the same way. EV-D amplicons were sequenced with the EVD_1D_S2 and EVD_1D_R2 primers described above. Molecular typing of HAV and HEV was performed using the VP1/2A junction and the ORF2 gene, respectively, by previously described methods [12,13]. The nucleotide sequences were obtained by standard Sanger sequencing. The nucleotide sequences obtained from environmental (n = 6 EV-D68; n = 6 HEV; n = 6 HAV) and clinical samples (n = 16 HEV; n = 4 HAV; n = 2 EV-D68) were deposited in the European Nucleotide Archive under accession numbers LT745908 to LT745947. Fifteen of the 17 EV-D68 sequences from clinical samples included in this study had been submitted previously [14].

### Sequence datasets and phylogenetic analysis

The nucleotide sequences from wastewater were compared with reference sequences representing the genogroups or genotypes known for each virus analysed and with the clinical sequences. These sequences were analysed by the neighbour-joining method (genetic distance calculated with the Tamura-Nei model) implemented in the MEGA 5 programme [15]. The confidence of phylogenetic relationships was assessed with 1,000 bootstrap replications. The classifications recently reported for EV-D and HEV were used to assign nucleotide sequences to genogroups or genotypes [9,16,17].

### Cell culture

Fifteen concentrated samples of wastewater taken during the year of sampling (n = 14 raw, n = 1 treated) were subjected to vigorous chloroform treatment (final concentration: 25%) for 1 min and centrifuged at 3,000 × g for 15 min. The upper phase was harvested and 400 µL were used to test virus infectivity on A549 cells (human lung carcinoma) grown in Dulbecco's Modified Eagle's medium supplemented with 5% fetal bovine serum, 1% of penicillin (10,000 U) and 1% streptomycin (10 mg/mL). Cytopathic effect was monitored daily for 7 days by microscopic examination. Four passages were performed for all samples. The primary identification of viruses was the appearance of cytopathic effects in cell monolayers, later confirmed by ADV and EV PCR (see above) according to cell line susceptibility.

## Results

### Virus distribution in raw wastewater

**Figure 1:**
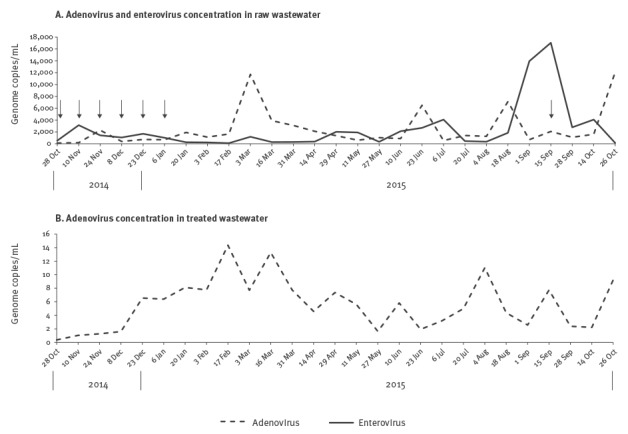
Dynamics of adenovirus and enterovirus concentrations over time in treated and untreated wastewater samples, Clermont-Ferrand, October 2014– October 2015 (n = 54 samples)

Internal controls added to all samples were correctly amplified. Thus, inhibitors were not detected in any of the concentrates processed from the 27 raw wastewater samples. All samples tested positive for ADV, EV, PeV, NoV (GI and GII genogroups) and RV genomes (Table 2). HAV and HEV genomes were detected in 16 of 27 and 10 of 27 samples, respectively, without seasonal distribution (Table 2). The median EV load (number of genome copies per mL of sample before concentration) was 1,177 (range: 102–17,046) copies/mL (Figure 1A). The EV load peaked in September 2015. A median viral load of 1,354 (range: 114–12,080) copies/mL was determined for ADV (Figure 1A). The highest ADV loads were detected in four samples obtained on 3 March, 23 June, 18 August and 26 October 2015.

**Table 2:** Virus detection in raw and treated wastewater waters, Clermont-Ferrand, October 2014– October 2015 (n = 54 samples)

Virus	Samples	2014					2015																					
		28 Oct	10 Nov	24 Nov	08 Dec	23 Dec	6 Jan	20 Jan	3 Feb	17 Feb	3 Mar	16 Mar	31 Mar	14 Apr	29 Apr	11 Maj	27 Maj	10 Jun	23 Jun	6 Jul	20 Jul	4 Aug	18 Aug	1 Sep	15 Sep	28 Sep	14 Oct	26 Oct
ADV	Raw	+	+	+	+	+	+	+	+	+	+	+	+	+	+	+	+	+	+	+	+	+	+	+	+	+	+	+
	Treated	+	+	+	+	+	+	+	+	+	+	+	+	+	+	+	+	+	+	+	+	+	+	+	+	+	+	+
RV	Raw	+	+	+	+	+	+	+	+	+	+	+	+	+	+	+	+	+	+	+	+	+	+	+	+	+	+	+
	Treated	ND	+	+	+	+	+	+	+	+	+	+	+	+	+	+	+	+	+	+	+	+	+	+	+	+	+	+
NoV	Raw	+	+	+	+	+	+	+	+	+	+	+	+	+	+	+	+	+	+	+	+	+	+	+	+	+	+	+
	Treated	ND	+	+	+	+	+	+	+	+	+	+	+	+	+	+	+	+	+	+	+	+	+	+	+	+	+	+
EV	Raw	+	+	+	+	+	+	+	+	+	+	+	+	+	+	+	+	+	+	+	+	+	+	+	+	+	+	+
	Treated^a^	+	+	-	+	+	+	+	+	+	+	-	+	-	+	+	-	+	+	+	+	+	+	+	+	+	-	-
HAV	Raw	-	+	-	+	-	+	+	-	-	-	+	+	+	-	-	+	+	-	+	-	+	-	+	+	+	+	+
	Treated	ND	-	-	-	+	+	-	-	-	-	-	-	-	-	-	-	-	-	-	-	-	-	+	-	-	+	+
HEV	Raw	-	-	-	-	-	+	-	+	-	-	-	-	-	-	+	-	+	+	-	+	+	-	+	-	+	-	+
	Treated	ND	-	-	-	-	-	-	-	-	-	-	-	-	-	-	-	-	-	-	-	-	-	-	-	-	-	-
PeV	Raw	+	+	+	+	+	+	+	+	+	+	+	+	+	+	+	+	+	+	+	+	+	+	+	+	+	+	+
	Treated	inh	+	+	+	inh	+	inh	inh	inh	+	inh	inh	-	inh	-	-	-	-	inh	-	-	+	-	+	-	-	+

ADV: adenovirus; EV: enterovirus; HAV: hepatitis A virus; HEV: hepatitis E virus; inh: inhibited samples after dilution (1/5 and 1/10); ND: not determined because of insufficient volume; NoV: norovirus; PeV: parechovirus; RV: rotavirus.

(+) presence and (−) absence of viruses as determined by qualitative or quantitative real-time (RT) PCR.

^a^ Detection by the qualitative EV PCR.

Virus concentrations obtained by quantification of genome copies in concentrated waters are expressed as the ratio of the initial sampling volumes and expressed in genome copies per mL. Arrows show dates of detection of EV-D68 in raw wastewater.

### Virus distribution in treated wastewater

All samples tested positive for ADV, RV, and NoV (Table 2). The median ADV load was 5.6 (range: 0.4 to 14) copies/mL. We calculated the reduction in ADV load between raw and treated wastewater at each sampling date. The mean reduction estimated was 3.14 ± 0.48 log_10_ copies/mL (Figure 1B). NoV GII was detected throughout the year in treated wastewater. NoV GI was detected in 15 of 26 samples that could be processed and was not identified during the summer. EV was not detected with the in-house RT-qPCR assay. In contrast, 21 of 27 samples tested positive with the qualitative assay. HAV was detected in five of 26 samples. 

Inhibitors were detected with the PeV and HEV RT-PCRs in samples of treated water (Table 2). An empirical dilution of 1/5 or 1/10 removed the inhibitors for the HEV test but did not for the PeV test in nine of 27 samples. Finally, HEV was never detected in treated wastewater and PeV genomes were present in eight of the 18 non-inhibited samples. 

### Viral infectivity

Cytopathic effects were observed after one to four passages in A549 cells for 10 of the 15 samples tested (raw wastewater: n = 9/14; treated wastewater: n = 1/1), which indicated that viruses were infectious after the concentration process. Consistently with viral cell tropism, the supernatants of cell cultures were tested for EV and ADV genomes by PCR. Five of 10 samples tested positive for both viruses, while five tested positive only for ADV.

### Circulation of enterovirus D68

**Figure 2:**
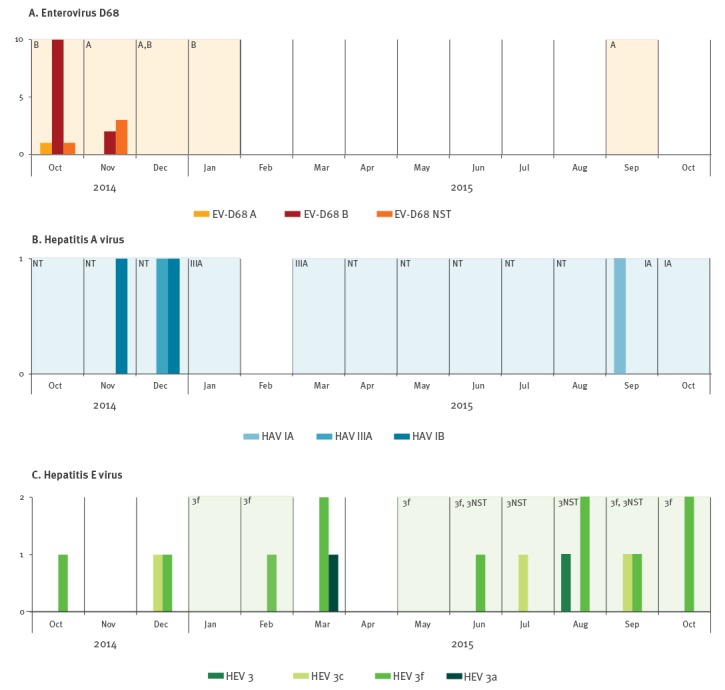
Comparison of temporal distribution of enterovirus D68, hepatitis A and E viruses detected in clinical (n = 37) and wastewater samples (n = 27), Clermont-Ferrand, October 2014– October 2015

EV: enterovirus; HAV: hepatitis A virus; HEV: hepatitis E virus; NT: non typable; NST: non subtypable.

**Figure 3:**
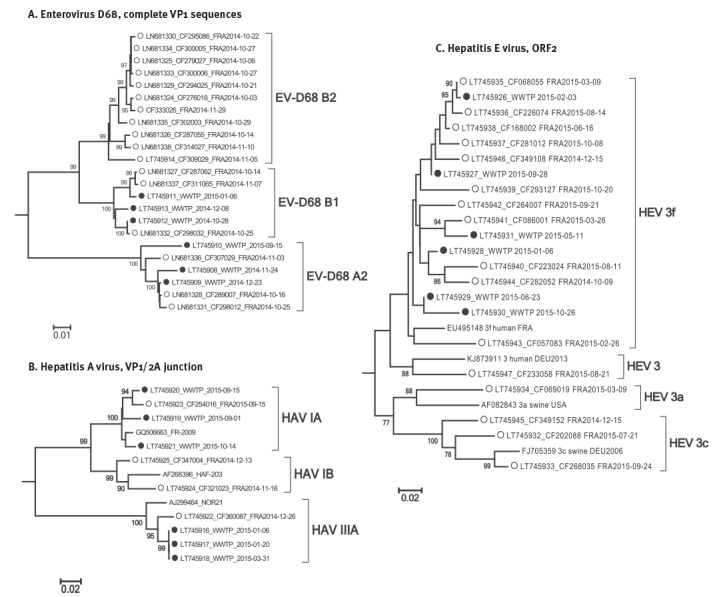
Phylogenetic trees of clinical and wastewater-derived sequences, Clermont-Ferrand, October 2014– October 2015 (n = 55)

EV: enterovirus; HAV: hepatitis A virus; HEV: hepatitis E virus;

Seven of 27 raw wastewater samples tested positive for EV-D by RT-PCR (Figure 1A). Six were collected between October 2014 and January 2015 and one in October 2015. EV-D68 was identified in the seven samples by sequencing. Nucleotide sequencing provided evidence of a mixture of several EV-D68 sequences in one sample (10 Nov 2014), which was excluded from the analysis. The nucleotide sequences were compared with those recovered in 17 patients hospitalised in October and November 2014 with respiratory illness (Figure 2A). No additional clinical cases were recorded between December 2014 and October 2015. Environmental sequences clustered in the EV-D68 clades A2 and B1 (Figure 3A). The environmental sequences detected between October 2014 and January 2015 showed close genetic (0.1–1.4% nucleotide difference) relationships with the EV-D68 clinical sequences of the same period. One isolate (WWTP_2015–09–15) assigned to clade A2 was unrelated to clinical cases (> 3% nucleotide difference).

Coloured background: virus detection in wastewater water over time. Vertical bars: number of concomitant clinical cases in the Clermont-Ferrand hospital. Genotyping results are indicated at the top.

Empty circles: clinical sequences, full circles: wastewater sequences, obtained during the period from October 2014 to October 2015. Only nodes supporting bootstrap values higher than 70% are represented. Trees were rooted with other clades: prototype strain Fermon (AY426531) for EV-D68, genotype 4 (FJ763142) for HEV and genotype V (D00924) for HAV (not shown).

### Hepatitis A and E viruses

HAV-positive wastewater samples were detected during the entire study period except in February 2015 (Figure 2B). Four acute HAV infections were diagnosed and notified during the same period. Of the four patients involved, three had recently returned from a trip abroad. The clinical strains were identified as HAV genotypes IA (n = 1, travel to Morocco), IB (n = 2, one patient with travel to Benin, one without travel history), and IIIA (n = 1, travel to India). HAV molecular typing was unsuccessful in 10 of 16 raw and five of five treated wastewater samples because the viral loads assessed with the Ct values of the RT-PCR assays were too low. Of six sequences analysed, three were assigned to genotype IIIA in samples collected in January and March 2015. These three sequences showed 2.5% nucleotide difference with that identified from a patient admitted in December 2014. The other three HAV genomes were collected in September and October 2015. They were assigned to genotype IA together with one patient infected during the same period with a closely related virus (1.7% nucleotide difference) (Figure 3B). The HAV genotype IB viruses identified in patients had no genetic relatives in the environmental samples (> 7% nucleotide difference).

Ten raw wastewater samples tested positive for HEV. Acute hepatitis E was confirmed in 16 patients who had no travel history, and hence the infections were autochthonous. There was no overlap between the temporal distribution of HEV-positive wastewater samples and clinical infections: clinical infections without wastewater detection occurred in October, December and March; wastewater detection without clinical infections occurred in January and May (Figure 2C). The phylogenetic tree topology obtained from the ORF2 region showed that all HEV strains belonged to genotype 3 (Figure 3C). Clinical strains were assigned to subtypes 3f (n = 11), 3c (n = 3), 3a (n = 1) and an as yet undefined subtype 3 (n = 1); the six wastewater sequences were identified as 3f (Figure 3C). HEV genotype 3 was identified in four other wastewater samples but they were excluded from phylogenetic analysis because they were too difficult to interpret (mixture of several sequences). The phylogenetic relationships between wastewater and clinical HEV subtype 3f sequences were heterogeneous (from 0.2% nucleotide difference between WWTP_2015–02–03 and LT745935 isolated one month later, to > 7% difference for WWTP_2015–10–26 with the nearest clinical sequence), a pattern which suggests distinct infections (Figure 3C). For example, three different viruses were identified in October 2015 (subtype 3f), and three others in March 2015 (subtypes 3f and 3a).

## Discussion

The method of wastewater concentration before viral detection was developed by combining techniques and processes used in two fields of microbiology. Tangential flow ultrafiltration, based on size exclusion, has successfully been used in aquatic microbiology studies. The documented advantages over classical filtration techniques range from the recovery of various microorganisms to rapid processing of large sample volumes even in highly turbid samples [18]. In our study, 20 L of influent or 50 L of effluent from the WWTP were processed in less than 1 hour without filter clogging. The concentration steps were optimised to overcome the usual aggregation and adsorption of viruses to organic particles as seen in wastewater and sediment matrices [19,20]. We did not determine the sensitivity of each virus recovery by the complete concentration process. However, the method has been reported as effective and conservative for virus concentrations from environmental water, sediments and wastewater [21-23]. The concentration and purification processes before molecular virus detection did not last more than 2 days. In addition, cell culture testing provided evidence that EV and ADV remained infectious in the concentrated samples processed in our study. This method could therefore be used to isolate and characterise an epidemic or a specific strain or variant previously detected by rapid molecular testing in a specific surveillance programme or a health alert.

Molecular testing was performed for seven enteric viruses. Of the 401 molecular tests performed in the study, only nine (2.2%) were unsuccessful because of PCR inhibitors in treated wastewater. This was unexpected in treated water but could be explained by the use of ferric chloride, a PCR inhibitor [24], during the wastewater treatment process implemented in this WWTP, only the PeV assay was affected. This could be explained by the differing sensitivity of polymerases to inhibitors [25]. In our sampling, all raw and treated wastewaters contained viruses. As expected, the number of viral pathogens detected per sample was lower in treated waters (mean: 4; range: 3–6) than in raw waters (mean: 6, range: 5–7). We compared our results with those reported in other recent European studies based on molecular detection of enteric viruses in wastewater. While these discordant results should be interpreted with caution because they could be due to epidemiological variations between countries and the diversity of methods used for virus recovery, our detection rates were similar to or higher than those previously reported (Table 3).

**Table3:** Detection rate of enteric viruses in different studies across several European countries, 2000–2015

Virus	Country	Reference	Collection year	Positive/total samples (%)	
				Influent	Effluent
ADV	France (Central France)	This study	2014–2015	27/27 (100)	27/27 (100)
	France (Paris)	[31]	2014	ND	100/100 (100)
	Italy	[29]	2013	17/21 (81)	7/21 (33)
	Italy	[30]	2010	24/25 (96)	19/25 (76)
RV	France (Central France)	This study	2014–2015	27/27 (100)	26/26 (100)
	France (Paris)	[31]	2013–2014	ND	86/100 (86)
	Italy	[37]	2010–2011	325/546 (60)	ND
NoV	France (Central France)	This study	2014–2015	27/27 (100)	26/26 (100)
	France (Paris)	[31]	2014	ND	98/100 (98)
	Italy	[38]	2007	62/64 (97)	26/33 (79)
	The Netherlands	[39]	2000–2001	23/25 (92)	26/28 (93)
EV	France (Central France)	This study	2014–2015	27/27 (100)	21/27 (78)
	France (Paris)	[31]	2014	ND	64/100 (64)
	Italy	[29]	2013	13/21 (62)	3/21 (14)
	Italy	[30]	2010	24/25 (96)	21/25 (84)
	United Kingdom (Scotland)	[35]	2009–2010	37/40 (93)^a^	ND
PeV	France (Central France)	This study	2014–2015	27/27 (100)	8/18^b^ (44)
	The Netherlands	[34]	2010–2011	28/89 (31)	ND
	United Kingdom (Scotland)	[35]	2009–2010	31/40 (78^a^)	ND
HAV	France (Central France)	This study	2014–2015	16/27 (60)	5/26 (19)
	France (Paris)	[31]	2014	ND	0/100 0)
	Italy (Piedmont)	[29]	2013	7/21 (33)	4/21 (19)
	Spain (Barcelona)	[40]	2006–2008	1/32 (3)	ND
HEV	France (Central France)	This study	2014–2015	10/27 (37)	0/26 (0)
	France (Paris)	[31]	2014	ND	0/100 (0)
	United Kingdom (Scotland)	[35]	2014–2015	14/15 (93)	ND
	Italy	[29]	2013	1/21 (5)	0/21 (0)
	Spain (Barcelona)	[40]	2006–2008	9/32 (28)	ND

ADV: adenovirus; EV: enterovirus; HAV: hepatitis A virus; HEV: hepatitis E virus; ND: not determined; NoV: norovirus; PeV: parechovirus; RV: rotavirus.

^a^ Solid waste.

^b^ Inhibited samples were excluded.

### Adenovirus, rotavirus, norovirus

ADVs, RVs and NoVs are three major gastroenteritis agents that persist in the environment from the discharge of treated wastewaters. They are recovered in surface, recreational and drinking water and can cause waterborne or food-borne disease outbreaks [26-28]. Our study showed the continuous presence of these three viruses over the year of surveillance in both raw and treated wastewaters, with 100% of tested samples positive. These recovery rates were equivalent to or slightly higher than those reported in other European studies and reflect wide virus circulation in the community. ADV loads were reduced by 3 log_10_ viral genome copies by the current treatment process, a result consistent with recent data from Italy [29]. The presence of infectious ADV particles in both influents and effluents showed that these viruses were not completely removed and/or inactivated by the current treatment of wastewater.

### Enterovirus

EVs are frequently detected in wastewater via the environmental surveillance of poliovirus implemented in several countries. The EV recovery rates are heterogeneous in Europe and range between 62 and 96% for raw wastewaters and between 14 and 84% for treated waters (Table 3). In our study, EV genomes were detected in 100% and 78% of raw and treated samples, respectively. In addition, infectious particles were present in 33% of samples inoculated into cell culture (raw and treated waters). The continuous detection of EVs in wastewater showed their sustained circulation in the community outside the epidemic period. Earlier studies reported an ADV genome concentration higher than that of EV in raw water samples [30,31], a pattern not found in our study. This result could reflect differences in the efficacy of the virus concentration process. The fact that EV was detected throughout the study period and not only during summer and autumn lends weight to this hypothesis.

### Enterovirus D68

The first European case of acute flaccid paralysis following EV-D68 pneumonia was reported in a child referred to the University Hospital of Clermont-Ferrand in September 2014 [8] during a period of epidemic circulation in Europe [9] and North America [10]. Because of the particular epidemiological context, we decided to focus our investigation on this emerging EV that is potentially associated with severe clinical presentations. EV-D68 has characteristics in common with rhinoviruses (it is acid-labile and mainly associated with respiratory symptoms) and is rarely detected in stools [32]. However, our study confirmed its detection in wastewater, as recently reported in Israel [33]. Between July and November 2014, the virus was also associated with respiratory infections, mainly in children admitted to the University Hospital of Clermont-Ferrand for asthma exacerbation or bronchiolitis (data not shown). Further cases were not seen until after May 2016 but our wastewater monitoring had evidenced silent circulation of EV-D68 in the community in the Clermont-Ferrand area in 2015. The present study shows that wastewater analysis is highly sensitive in tracing viral circulation in the area connected to the WWTP and in detecting the co-circulation of several distinct viral lineages.

### Parechovirus

PeV infections, often clinically indistinguishable from EV infections, are increasingly identified but their burden is probably underestimated. Two previous European studies reported their presence in raw wastewater at different sampling points in the Netherlands [34] and in solid waste samples in Edinburgh, Scotland [35] (Table 3). We found a positive detection rate of 100% and 44% in raw and treated wastewaters, respectively, indicative of sustained viral circulation in the local community.

### Hepatitis A virus

Like other European countries, France is currently considered to be of low endemicity for HAV infections because of the general high quality of wastewater collection and treatment installations. However, we found higher detection rates in raw wastewater (60%) than those reported in Spain (3%) and Italy (33%), although this is based on few samples (Table 3). Although few symptomatic acute HAV infections have been notified in our area, wastewater monitoring has evidenced a sustained circulation of the virus in the community. The pathogen has been mainly associated with asymptomatic infections but overflows or wastewater releases could still be a risk of HAV contamination in a population with low herd immunity.

### Hepatitis E virus

To our knowledge, this is the first prospective study monitoring the presence of HEV in wastewater in France. HEV was the only virus not detected in treated waters and the least prevalent in raw wastewaters (37% of samples tested). Similar results were reported in Spain but the rates of positive samples differed in other countries in raw wastewater (5% in Italy and 93% in Scotland, Table 3). In our study, clinical and wastewater sequences were only distantly related. The diversity of phylogenetic lineages suggests the co-circulation of multiple lineages within genotype 3 and multiple sources of contamination in the general population. Further studies will be necessary to assess the role of environmental transmission in the epidemiology of HEV in Europe, where zoonotic transmission from consumption of raw or undercooked meat is the main cause of the circulation of HEV genotype 3 in the population [36].

## Conclusion

The method developed in this study, based on a high performance concentration process combined with molecular detection, was able to monitor in real time the circulation of a large panel of human enteric viruses in urban wastewater during environmental surveillance. The comparison of clinical and wastewater-derived sequences could provide a more detailed picture of the epidemiology of infections in the local community.
